# The Potential Role of Lycopene for the Prevention and Therapy of Prostate Cancer: From Molecular Mechanisms to Clinical Evidence

**DOI:** 10.3390/ijms140714620

**Published:** 2013-07-12

**Authors:** Nina Pauline Holzapfel, Boris Michael Holzapfel, Simon Champ, Jesper Feldthusen, Judith Clements, Dietmar Werner Hutmacher

**Affiliations:** 1 Regenerative Medicine, Institute of Health and Biomedical Innovation, Queensland University of Technology, 60 Musk Avenue, Kelvin Grove, QLD 4059, Brisbane, Australia; E-Mails: ninapauline.holzapfel@qut.edu.au (N.P.H.); holzapfel@orthopaedic-oncology.net (B.M.H.); 2 Human Nutrition, BASF SE, G-ENH/MB, 68623 Lampertheim, Germany; E-Mails: simon.champ@basf.com (S.C.); jesper.feldthusen@basf.com (J.F.); 3 Australian Prostate Cancer Research Centre, Translational Research Institute, 37 Kent Street, Woolongabba, QLD 4102, Brisbane, Australia; E-Mail: j.clements@qut.edu.au; 4 The George W. Woodruff School of Mechanical Engineering, Georgia Institute of Technology, 801 Ferst Drive Northwest, Atlanta, GA 30332, USA; 5 Institute of Advanced Study, Technical University of Munich, Lichtenbergstr. 2a, 85748 Garching, Munich, Germany

**Keywords:** lycopene, prostate cancer, disease prevention, treatment, molecular mechanisms, animal models

## Abstract

Lycopene is a phytochemical that belongs to a group of pigments known as carotenoids. It is red, lipophilic and naturally occurring in many fruits and vegetables, with tomatoes and tomato-based products containing the highest concentrations of bioavailable lycopene. Several epidemiological studies have linked increased lycopene consumption with decreased prostate cancer risk. These findings are supported by *in vitro* and *in vivo* experiments showing that lycopene not only enhances the antioxidant response of prostate cells, but that it is even able to inhibit proliferation, induce apoptosis and decrease the metastatic capacity of prostate cancer cells. However, there is still no clearly proven clinical evidence supporting the use of lycopene in the prevention or treatment of prostate cancer, due to the only limited number of published randomized clinical trials and the varying quality of existing studies. The scope of this article is to discuss the potential impact of lycopene on prostate cancer by giving an overview about its molecular mechanisms and clinical effects.

## 1. Introduction

Prostate cancer is the second most frequently diagnosed cancer worldwide among men. Incidence rates are highest in Australia, Western Europe and Northern America, whereas the lowest incidence rate is found in South-Central Asia. In 2008, prostate cancer was the sixth leading cause of death from cancer among men worldwide. Prostate specific antigen (PSA) testing has been shown to have a greater influence on incidence than on mortality [1]. Therefore, there is a larger variety in incidence rates than in mortality rates. Interestingly, the number of deaths in developing countries is not excessively different from more affluent countries [2]. This fact generated a discussion in the field about the benefit of early detection and aggressive treatment of early localized prostate cancer [3]. While in many cases, there is still uncertainty about the time of initiation and the radicalness of the therapy, the field agrees about the need for effective preventive measurements of the disease [4].

In the last decade, an increasing amount of scientific information has been published about the potential to prevent diseases by complementary and alternative medicine [5]. These studies might influence people’s attitude towards the use of dietary supplements, but there is also a rising interest of consumers in their own healthcare [6]. These so-called “community-based lifestyle interventions” are envisioned to improve general health, boost the immune system and even provide a cure for cancer. Recent studies have shown that prostate cancer patients are particularly attracted by complementary or alternative medicine products [7,8]. This is easy to understand when one is made aware of the fact that incidence rates of prostate cancer vary by more than 25-fold worldwide [2], suggesting that environmental or dietary factors might play a role in the development of prostate cancer [9]. The prevalence of using complementary and alternative therapeutic methods among patients with prostate cancer has been shown to be around 30% [7], and approximately 25% use herbs, minerals, vitamins and antioxidants, such as green tea, selenium, vitamin E or lycopene [10]. Patients with advanced forms of the disease and those treated with hormones are even more susceptible to use these products [8].

The interest on lycopene rich diets and supplements for the prevention or therapy of prostate cancer has extremely increased during the last years. Lycopene products are well tolerated and meet the requirements of the US Food and Drug Administration for the designation of Generally Recognized as Safe (GRAS). However, it is still questionable if there is a definitive evidence to recommend it to patients in addition to a well-balanced diet. The aim of this review is to give an up-to-date overview about the properties of lycopene, its molecular and cellular mechanisms with special regard to the actual clinical evidence for its use in the prevention and therapy of prostate cancer.

## 2. Chemical Properties of Lycopene

More than 600 carotenoids are known to be naturally occurring. These predominantly colourful molecules, built by plants, fungi and bacteria undergoing photosynthesis, are widespread in vegetables and fruits [11,12]. Carotenoids are divided into two main groups. Of them, highly unsaturated hydrocarbons consisting of lycopene, α-, β-, and γ-carotene build the first group, whereas xanthophylls, such as β-cryptoxanthin, lutein, and zeaxanthin, are considered as the second big carotenoid-group. The first class, hydrocarbon carotenoids, contains only carbon- and hydrogen-atoms, but lack oxygen, whereas xanthophylls, in contrast, consist of at least one oxygenated group on their terminal rings [12]. Even though all carotenoids exhibit certain joint chemical features, like a distinct conjugated double bond system, a polyisoprenoid structure and an almost bilateral symmetry around the centrical situated double-bond, modifications of the main structure, like cyclization of terminal groups and insertion of oxygen functions, lead to different types of carotenoids with varying characteristic colours and antioxidant qualities [13,14].

**Figure 1:**
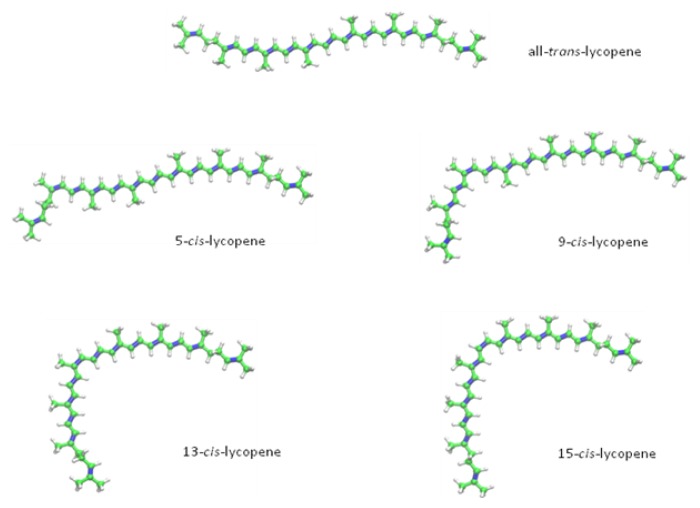
All-trans-lycopene and its metabolites, 5-*cis*, 9-*cis*, 13-*cis* and 15-*cis*-lycopene.

Lycopene, a representative of the hydrocarbon carotenoids with the molecular formula of C_40_H_56_, has an acyclic open-chain structure consisting of 13 double-bonds. Two of them are non-conjugated, whereas eleven are conjugated double bonds, thereby building a chromatophore [12]. This distinctive conjugated polyene structure accounts for the ruby colour and the antioxidant properties of lycopene [12]. It has a distinct lipophilic character, which makes it nearly insoluble in ethanol, methanol and water [15]. Due to its acyclic structure and the absence of a β-ionone ring, there is no pro-vitamin A activity to be found in lycopene, which is the reason for its differing biochemistry, as compared to α- and β-carotene [15]. The prevalent biological occurring form of lycopene is the all-*trans*-isomer, which is also the thermodynamically most stable configuration [16]. Indeed, heat, light or several chemical reactions can induce isomerization from the *trans*-isomer to various mono- or poly-*cis* forms [12]. The lycopene forms detected in human serum and tissues range from all-*trans* to 9-, 13- and 15-*cis* isomers, whereas the predominant naturally occurring configuration in food is the all-*trans* form (Figure 1). Hence, it is expected that *in vivo* isomerization mechanisms exist [17].

### 2.1. Anti-Oxidant Properties

Due to its polyene-structure, providing an electron-rich system, lycopene is an eligible target for electrophilic reagents. Thus, it performs an uttermost reactivity towards oxygen and free radicals [18]. Lycopene is known to be the most potent oxygen quenching reagent among carotenoids, and furthermore, it provides the ability to intervene in reactions initiated by free radicals, like OH^−·^ or peroxy radicals [18,19]. Its excellent anti-oxidant properties are most likely the basis for its preventive role towards cancer and other chronic diseases.

The ability to quench oxygen increases with the opening of the β-ionone ring in the molecule structure, as found by direct comparison of lycopene, γ-carotene and β-carotene. This might be the result of the lowering of the energy level, which provides the ability to approach a triplet energy level. From this stage, the energy transfer from the excited state of oxygen can be adopted easily [19]. As lycopene exhibits the greatest potential in oxygen quenching among all carotenoids, its isomers have been found to vary in their antioxidant properties, as well. 5-*cis* lycopene has been found to be most potent, followed by 9-*cis*. The weakest antioxidant properties have been reported for the all-*trans* isomer [20].

Carotenoids or lycopene, respectively, act as antioxidants through several mechanisms. Highly reactive oxygen species, also named singlet oxygen (^1^O_2_), which are able to oxidize nucleic acids, unsaturated fatty acids or amino acids, can be quenched by carotenoids/lycopene exerting the reaction stated below [18]:

O12+LYC→O32+L3YCL3YC→LYC+heat

The exceeding amount of energy, the lycopene molecule gained in this reaction reaching the triplet state, is dispensed through vibrational, as well as rotatory interactions with the solvent, resulting in the release of thermal energy. Once more, the extensive conjugated polyene structure of lycopene is responsible for this reaction. As the molecule re-establishes its ground state immediately, another ^1^O_2_ quenching cycle can be activated, thereby providing the possibility of each single carotenoid-molecule to quench about 1000 molecules of ^1^O_2_[18].

Lycopene and other carotenoids are also known for their antioxidant activities towards impeding free radical reactions. Peroxyl radicals are built in the organism during the process of lipid peroxidation, which can lead to destruction of lipophilic sections. Inactivation of these reactive species results in the development of radical adducts that build a resonance-stabilized carbon-centred radical. The carotenoid oxidation products include formation of epoxides located at the β-ionone ring, as well as located at the central double bond of the conjugated polyene chain. More products of this reaction are the formation of ketones and aldehydes at the β-ionone ring [21]. Inhibition of these radical reactions by lycopene may shelter membranes from lipid peroxidation [22].

Furthermore, lycopene is in discussion to be able to repair vitamin E and C radicals *in vivo* by conducting the following reaction [23]:

$$\text{LYC}+\alpha -\text{Tocopherol}+{\text{H}}^{+}\to {\text{LYC}}^{+}+\alpha -\text{Tocopherol}-\text{H}$$

Besides its radical reactions, lycopene has also been shown to upregulate the so-called antioxidant response element (ARE). Cellular enzymes, like glutathione S-transferase, superoxide dismutase or quinone reductase, are activated by lycopene, resulting in another way of protecting cells against highly reactive oxygen species [15]. Linnewiel *et al*. reported that hydrophilic oxidation products of carotenoids, rather than the intact lipophilic carotenoid molecules, have been found to be responsible for the stimulation of the ARE system, detected *in vitro* using LNCaP and MCF-7 cells [24]. Oxidized lycopene derivatives, built due to the instability of these molecules, are present in tomatoes, as well as in human serum and tissues. However, these oxidative products have been found to be present in different human tissues only at a rather small amount as compared to the parent compound. The described oxidation is most likely a natural metabolism reaction in tomatoes or can also happen during heat-processing of tomato-rich foods. Furthermore, *in vivo* oxidation reactions in humans resulting in the development of lycopene epoxides are discussed in the literature [25]. However, the contribution of these lycopene derivatives as active antioxidant compounds might be insignificant *in vivo*, due to the predominance of the intact lycopene molecule in most human tissues.

### 2.2. Lycopene-Content in Different Sources

Natural sources of lycopene include *inter alia* watermelon, rosehips, pink grapefruit, guava, apricots and, above all, tomatoes (Table 1).

As all tomato products contain high concentrations of lycopene, they are, at the same time, the most important source of this carotenoid for humans, accounting for over 85% of all dietary sources [26]. However, the lycopene concentration in fresh fruits shows a great variability, depending on environmental conditions, geographic location, climatic situation, species and maturity, but with an average of about 5 to 10 mg lycopene per 100 g tomato [12,28]. Up to 15 mg lycopene in 100 g fruit has been found for deep-red tomato varieties, whereas yellow species are less rich in lycopene, with a content of only about 0.5 mg per 100 g [28].

Attempts to compare lycopene contents between organic grown tomatoes and conventionally grown ones emerged to be not consistent. Rossi *et al*. and Hallmann *et al*. found higher contents of lycopene in conventionally grown fruits [29,30], whereas Caris-Veyrat *et al*., on the contrary, discovered the opposite. They found a significantly higher amount of lycopene in organic grown tomatoes [31]. These differences could be appropriate to the findings of Ordonez-Santos *et al*. reported in 2011. They didn’t find statistically significant differences between organic and conventionally grown tomatoes, but reported that the main factor affecting the micronutrient content of tomatoes might be the cultivar, rather than the cultivation method [32].

Processed and, therefore, less hydrated tomato-products have been found to be richer in lycopene than whole, raw tomatoes. Tonucci *et al*., for example, examined the lycopene content of different tomato-based foods, like whole tomatoes, ketchup, spaghetti sauce, tomato paste, tomato puree and tomato sauce, thereby finding great differences between the raw fruit and processed products (Table 2) [33].

### 2.3. Bioavailability and Metabolism of Lycopene

Absorption of lycopene from dietary sources occurs within the range of 10 to 30% in humans [34]. After ingestion, lycopene is taken up by dietary lipid micelles and incorporated into the mucosa of the small intestine. The micelles are packaged into chylomicrons, which are then transported to the liver using the lymph system. Lipoproteins carry the lycopene molecules into the plasma, from where they are distributed to their target organs [35]. Maximal concentrations have been found in the testes, prostate, adrenal glands and liver [36].

Many lifestyle factors, like smoking, consumption of alcohol, blood lipid levels, and also biological factors, such as age or hormonal status, have an influence on the absorption of lycopene [36]. Studies have also shown that absorption from processed tomato products is better than from raw tomatoes. Stahl *et al*. found an increase in the level of serum lycopene in humans only after consumption of processed juice, but not after administering unprocessed tomato juice [34]. Gaertner *et al*. compared tomato paste with fresh tomato salad, thereby finding a twofold increase of lycopene in serum after consumption of the paste [37]. As the bond between lycopene and macromolecules within the food-matrix is comparatively strong, its bioavailability from dietary sources is rather remote, but can be enhanced by processing the food, like cooking or chopping, in order to separate complexes between lycopene and protein [12,38]. The food matrix, to which lycopene is tightly attached, is thought to contribute to the maintenance of a stable all-*trans* isomer, thereby preventing the molecule to isomerize to the *cis*-conformation. By disrupting membranes of chromoplasts and reducing the integrity of cells, thermal and mechanical treatment may cause an easier release of lycopene from its surrounding matrix. The unbound molecule is now accessible for isomerisation into the *cis*-conformation, which is in discussion to improve the bioavailability [12,38]. Due to its lipophilic character, the addition of lipids to lycopene-containing foods can enhance the bioavailability, as well [39].

In most food sources, lycopene exists predominantly in the all*-trans* conformation. In contrast to this finding, the predominant isomeric form found in human and animal tissues—More precisely, >50% of total lycopene, is the *cis*-isomer [17]. One of the reasons for this difference is that the *cis-*isomer is thought to provide a better bioavailability and that it might be more easily absorbed. Its solubility in the bile acid micelles is enhanced, due to a slightly changed structure, which makes the molecule shorter and, at the same time, more suitable to fit into the micelle [40,41]. All*-trans* isomers have been found to exhibit a greater disposition to aggregate in the intestine, thereby building crystals, which may reduce their uptake through micelles [14]. Other studies investigating the bioavailability of lycopene indicated that possible isomerisation procedures in the stomach could be the answer for the enhanced presence of *cis*-lycopene in human tissues, as compared to *trans*-lycopene [42,43]. Re *et al*. [42], for example, incubated lycopene from dietary supplement capsules or, alternatively, tomato puree with human or simulated gastric juice to detect the percentage of isomerisation. The amount of *cis*-isomers increased for both sample-types after treatment with gastric juice, but the dietary capsules showed a higher percentage in the *cis*-stereoisomeric form, which might be an indicator for the stabilizing effect of the plant matrix. Isomerisation was furthermore dependant on the pH level, as it was enhanced when the pH was below 2 and as it remained static when the pH was adjusted to ~7. Following these findings, the most supposable reason of *cis*-isomer formation might be the existence of an acidic environment in the stomach [42]. As the findings of lycopene bioavailability and absorption are still not entirely clear, there are more investigations required to detect and understand the pathways of its metabolism [44].

Due to a consistently increasing market for dietary supplements, studies have been conducted, investigating the bioavailability of synthetic lycopene as compared to naturally derived, unprocessed or processed lycopene. For this purpose, Hoppe *et al*. [45] used two different Lycopene beadlet formulations, which have been administered to 36 healthy volunteers over a period of 28 days: LycoVit™ 10% (BASF, Ludwigshafen, Germany, synthetic lycopene) and Lyc-O-Mato™ beads 5% (LycoRed Natural Products Industries Ltd, naturally derived lycopene). Lyc-O-Mato™ is a tomato-oleoresin, extracted from tomatoes. It contains lycopene and other phytonutrients, such as tocopherols, phytoene, phytofluene, β-carotene, phospholipids and phytosterols. The findings of this study resulted in a precise and similar increase of total, as well as *cis*- and *trans*-lycopene serum response for both sources, thereby indicating an equivalent bioavailability of synthetic and naturally-derived lycopene [45].

Cohn *et al*. [46] compared plasma kinetics after administering synthetic lycopene (5 mg lycopene tablets, 5%), a processed natural lycopene source (tomato soup from tomato paste) and an unprocessed natural source (tomato juice). In this study, the absorption of the synthetic formulation was comparable to the processed product, but enhanced as compared to the unprocessed tomato juice. This demonstrates again the importance of processing food to enhance the bioavailability of lycopene [46]. Results from a study performed by Tang *et al*. [47] demonstrate even a predominance in the bioavailability for synthetic lycopene as compared to the natural carotenoid from steamed-pureed tomatoes, as they found almost three-times higher lycopene-absorption in human probands of the synthesized product than for the natural one. The tomato matrix, as well as the preparation-procedure, which didn’t include heating in oil, might be responsible for this inferior outcome of the natural source [47].

The results of these findings might be of great interest, particularly for elderly and multi-morbid people, as their ingestion can be reduced or disturbed. Lycopene intake in the form of a supplement could most likely provide a more convenient way of benefitting from its potential antioxidant and anti-cancer properties for those patients than consuming lycopene-rich food. The potential properties of lycopene to target cancer cells will be discussed in the following sections.

## 3. Molecular Mechanisms of Lycopene on Prostate Cancer Cells: *In Vitro* Models

One of the possible reasons leading to prostate cancer development includes a series of mutations in genes controlling cell differentiation and growth. A chronic inflammation process in bacterial prostatitis, for example, can lead to the production of reactive oxygen species (ROS). This might contribute to oxidative damage of DNA, resulting in gene mutations. Exogenous carcinogens can have similar effects. DNA defects are usually detected by the cell surveillance system, which initializes cell cycle arrest to re-establish DNA integrity. If the DNA damage is irreparable, the consequence will be apoptosis of the cell. However, malfunctioning repair mechanisms can initiate mutations, followed by potential cancer development [48].

### 3.1. Prevention of DNA Damage

Androgen has been reported to induce an increase of ROS in prostate cancer cells [49]. Goo *et al*. investigated changes in protein-expression of androgen-depleted and androgen-sufficient LNCaP cells by conducting quantitative proteomic analysis [50]. After treatment with 0.2 μM lycopene, they found increased numbers of detoxification proteins, such as epoxide hydrolase-1, which is involved in the hydrolysis of epoxides to transfer these proteins to less reactive species. Furthermore, the proteins, superoxide dismutase-1, which degrades radicals in cells, and catalase, which protects cells from hydrogen peroxide, have been found to be increased. This has also been detected for metal binding protein, transferrin, which binds to iron and prevents it from producing oxidative stress. Lycopene has been found to induce a moderate increase of these detoxification proteins in androgen-sufficient and a significant increase in androgen-depleted LNCaP cells. Though the amount of detected proteins has been low, it can be hypothesized that lycopene provides a protective mechanism in preventing DNA damage, due to an increased expression of these proteins [50]. Another study conducted by Qiu *et al*. in 2013 analysed protein expression by using iTRAQ proteomics after exposure of prostate epithelial cells to 2 μmol/L lycopene for 48 hours. Lycopene increased the amount of phase II protective enzymes, such as glutathione-*S*-transferase-omega-1, peroxiredoxin-1 and sulphide-quinone oxidoreductase. Proteins, such as ERO1-like protein-α or CLIC-1, usually involved in ROS generation, have been shown to be downregulated after treatment with lycopene. This indicates a potential of lycopene to lower the risk for the generation of ROS and to reduce oxidative stress [51].

By using ferric nitrilotriacetate (FE-NTA) plus ascorbic acid, Matos *et al*. [52] induced oxidative damage to DNA in green monkey kidney fibroblasts (CV1-P), measured via the formation of 8-hydroxydeoxyguanosine (8-OHdG), which has been shown to be a specific marker for oxidative DNA damage. The presence of 8-OHdG has been discussed to be associated with mutagenesis and carcinogenesis [53,54]. Addition of lycopene in a concentration of 20 pmol/10^6^ cells has been shown to decrease 8-OHdG levels in DNA by 77%, thereby indicating a protective effect of lycopene against oxidation of DNA in mammalian cells [52].

However, a study carried out by Hwang *et al*. [55], investigating the protective potential of lycopene towards oxidative DNA damage in LNCaP human prostate cancer cells, revealed not only failure in protection against oxidative DNA damage at physiological concentrations of lycopene (0.1–1 μM), but also a pro-oxidative effect at higher lycopene concentrations (>5 μM) [55]. This has also been shown by Lowe *et al*. [56] in a study using a HT29 colon carcinoma cell line. Low doses (1–3 μM) of lycopene or β-carotene protected DNA from damage induced by xanthine/xanthine oxidase. However, by increasing the concentrations (4–10 μM) of the test substances, the opposite effect has been observed [56].

Anti-oxidant and pro-oxidant qualities of lycopene under cell culture conditions seem to change in a time- and dose-dependent manner. Pro-oxidant effects of lycopene have been observed at concentrations that are higher than physiological concentrations seen *in vivo* [55].

### 3.2. Effects on Tumour Cell Proliferation and Growth

Kotake-Nara *et al*. [57] examined 15 different carotenoids with regard to the potential of growth inhibition to the prostate cancer cell lines, PC3, DU145, and LNCaP. The authors found a significantly reduced cell viability after treatment with acyclic carotenoids at concentrations of 20 μmol/L Treatment at a concentration of 5 μmol/L resulted in an enhanced reduction of cell viability in particular for lycopene [57]. Another study, using a hexan extract of tomato paste resulted in a time- and dose-dependent decrease of cell proliferation of LNCaP cells, with maximal effects detected at a concentration of 5 μM lycopene. After 48 hours of incubation, growth inhibition reached 67%. However, as a tomato paste extract has been used instead of pure lycopene, it might be possible that the results have additionally been induced by other tomato components [58]. Ivanov *et al*. [59] compared two lycopene preparations, a 3% lycopene formulation from tomato powder (Lycopen™) and a tomato extract (LycoTrue™) to assess their potential to affect the proliferation of androgen-responsive LNCaP prostate cancer cells, as well as androgen-independent PC3 prostate cancer cells. They found a significant reduction in cell proliferation of LNCaP cells upon treatment with 1 μM or more Lycopen™. LycoTrue™ reduced cell numbers significantly at a concentration of 0.5 μM after 48 h. Cell proliferation was not affected after exposure for 24 h or with only 0.25 μM lycopene. Repeating the experiments with androgen-independent PC3 prostate cancer cells showed similar results. As the density of LNCaP cells has been found to be reduced after treatment for 48 hours and no change has been found for PC3 cell density before and after treatment, a cytotoxic effect of lycopene to LNCaP cells and a simply cytostatic effect of lycopene on PC3 cells has been suggested [59]. Another study, using DU145 cells, found that lycopene induced a reduction of the proliferation rate at concentrations of 15 and 25 μM, but not at physiological concentrations below 2 μM. To address if these effects have been due to oxidative products of lycopene, the study examined anti-proliferative effects of apo-12-lycopenal and apo-8-lycopenal (1–25 μM) on DU145 cells, thereby finding a significant reduction of proliferation only for apo-12-lycopenal. This finding indicates bioactivity for at least one of the lycopene metabolites [60]. In 2012, Yang *et al*. [61] showed that a lycopene induced activation of the PPARγ-LXRα-ABCA1 pathway was associated with anti-proliferative effects in LNCaP cells. The nuclear receptors, PPARγ and LXRα, build heterodimers with the retinoid X receptor. Activation of these heterodimers has been reported to be involved in growth inhibition of prostate cancer cells [62,63]. Treatment of LNCaP cells for 24–96 h with concentrations up to 10 μM lycopene revealed anti-proliferative effects, as well as an increase in protein- and mRNA-expression of PPARγ, LXRα and ABCA1 [61]. Another study conducted by this group was extended to the use of the androgen independent cell lines, DU145 and PC3, resulting in a similar outcome [64]. They indicated a relationship between the ability of lycopene to reduce proliferation through the PPARγ-LXRα-ABCA1 pathway with an increase in cholesterol efflux in the tested cell lines. As an increased cholesterol metabolism has been reported to be involved in cancer progression, this finding might also be indicative for the antitumor potential of lycopene [61,64].

Contradictory to the studies discussed above, Burgess *et al*. [65] reported a failure of lycopene in inhibiting growth of DU145 cells at concentrations between 0.0001 and 10 μM and over a time of 72 h. The lycopene concentrations used in this study have been reported to lie within the physiological range [65]. Liu *et al*. [66] investigated the potential of lycopene and apo-10-lycopenal in preventing prostate cancer growth through epigenetic modifications. More precisely, they investigated a possible potential of the carotenoid to vary methylation patterns of DNA using LNCaP cells in both physiological and supra-physiological concentrations and examined the ability of lycopene or its metabolites to recover the expression of glutathione *S*-transferase P1 (GSTP1) through demethylation. However, they didn’t find any differences in DNA methylation of the promoter of GSTP1, thereby indicating that lycopene and its metabolites are not effective in demethylating DNA of LNCaP cells and, correspondingly, that they don’t affect cancer growth by inducing epigenetic modifications [66].

### 3.3. Effects on the Cell-Cycle

The loss of the ability to regulate the cell-cycle is characteristic for cancer cells and results in uncontrollable proliferation. Processing cells through the first gap (G_1_) phase of the cell cycle is a step which is frequently disordered in cancer [67]. Lycopene has been investigated in different studies for its ability to mediate cell cycle arrest.

Hwang *et al*. demonstrated that a tomato-paste extract induced arrest in both the G_0_/G_1_ phase and the G_2_/M phase of the cell-cycle of LNCaP cells via flow cytometry. First, effects have been detected after 24 h with treatment of 0.5 μM of the extract and increased with higher concentrations and expanded time periods. These results indeed showed the efficiency of the tomato paste extract to inhibit tumour cell proliferation at physiological concentrations. However, the authors did not analyse what components of the extract have been responsible for this finding [58]. Another study examined the influence of lycopene and one of its metabolites, apo-12-lycopenal, on the cell-cycle of DU145 cells. The authors found a changed distribution pattern in the cell-cycle at concentrations 1–25 μM, with accumulation of cells in the G_1_ and G_2_/M-phase and reduction of cell numbers in the S-phase. Palozza *et al*. reported lycopene-induced cell-cycle arrest by describing molecular mechanisms [68]. After lycopene treatment, they revealed a reduced binding ability of NF-κB in LNCaP cells, which controls cell growth by influencing cell-cycle related proteins, and different apoptosis mediating proteins, like cyclin D1, p21, p27, p53, Bax or Bcl-2. A 24 hours exposure of lycopene resulted in a dose-dependent decrease of the G_0_/G_1_ phase-related protein, cyclin D1, and an increase in the cyclin kinase inhibitors, p53, p21 and p27. Measurement of cell-cycle distribution of LNCaP cells showed a clear increase in the number of cells in the G_0_/G_1_-phase and a corresponding decrease of the number of cells in the S-phase [68]. Furthermore, this study revealed that lycopene interferes with the mevalonate pathway by reducing the expression of HMG-CoA reductase and by decreasing intracellular cholesterol levels upon treatment with concentrations ≥2.5 μM. Prenylation and translocation of Ras, which might play a role in redox regulation, has been found to be decreased after 24 h treatment of LNCaP cells. Subsequently, this might lead to NF-κB activation via modulation of redox-sensitive pathways and induce the above mentioned cell cycle arrest [68].

### 3.4. Potential to Induce Apoptosis

Through the elimination of damaged, abnormal cells, the maintenance of a healthy, physiological organism is guaranteed. Cancer cells are, amongst others, characterized by the loss of the ability to undergo apoptosis [15].

Lycopene has been investigated for its capability to induce apoptosis in a couple of *in vitro* studies: Hwang *et al*. [58] detected apoptosis in the hormone sensitive prostate cancer cell line, LNCaP, after treatment with tomato paste extract for 24 and 48 h using an annexin V-FITC detection kit. Apoptosis has been detected predominantly in late stages, and most of the treated cells responded after 24 h of exposure to the tomato paste extract. Importantly, a significant increase in apoptosis has been reported after treatment with the physiologically relevant concentration of 1 μM [58]. Hantz *et al*. used pure lycopene instead of tomato extract to study its effect on apoptosis [69]. Alterations in mitochondrial function are a typical step in early apoptosis. These alterations involve enhanced permeability of the mitochondrial membrane to proteins, like cytochrome c, and disruption of the transmembrane potential [70]. Exposure of LNCaP cells to physiologically relevant concentrations of 0.3, 1.0, and 3.0 μM lycopene induced a dose-dependent pro-apoptotic effect. Mitochondrial function was reduced at all concentrations tested, and a significant decrease of the mitochondrial transmembrane potential has been detected at concentrations of 1 and 3 μM lycopene. Furthermore, the authors found an increase of cytochrome c in the fraction of lycopene-treated cells as further evidence of apoptosis [69]. Ivanov *et al*. [59] reported a significant increase of apoptosis in LNCaP cells upon treatment with 0.08 μM lycopene, reaching a steady state at 0.4 μM. However, concentrations of lycopene up to 0.8 μM failed to cause apoptosis in PC3 prostate cancer cells. Unlike LNCaP cells, PC3 cells are not hormone-sensitive. In order to determine if signalling of the androgen receptor might be involved in apoptosis-inducing processes, androgen-responsive luciferase reporter assays have been used in this study. These assays revealed that lycopene has no impact on the signalling of these receptors and that the different outcome of the apoptosis studies might therefore not be dependent on androgen signalling [59]. Using concentrations of 20–60 μM lycopene, Kanagaraj *et al*. [71] detected apoptosis in PC3 cells. This finding was based on an increasing amount of insulin-like growth factor binding protein-3 and increased binding of annexin V and propidium iodide [71]. However, these concentrations are not attainable under physiological conditions. Teodoro *et al*. [72] found increased apoptosis rates in DU145 prostate cancer cells after incubation with 3 μM lycopene for 96 h. This dose comes close to those found in humans [72]. In a proteomic study using primary prostatic epithelial cells instead of a prostate cancer cell line, treated with 2 μmol/L of a lycopene beadlet formulation for 48 h, Qiu *et al*. investigated lycopene-induced changes in the expression profile of proteins, thereby finding an upregulation of pro-apoptotic proteins (tyrosyl-tRNA synthetase, 40S ribosomal protein S3 and pyruvate kinase isozyme M2), as well as a downregulation of anti-apoptotic proteins (chloride intracellular channel protein 1, heat shock 70 kDa protein 1A/B, HSPβ1, Rho GDP-dissociation inhibitor 1, translationally controlled tumour protein, lactoylglutathione lyase, 78 kDa glucose-regulated protein and protein-kinase C inhibitor protein1) [51]. However, irrespective of changes in protein levels, there was no evidence of apoptosis in lycopene-treated primary prostatic epithelial cell cultures [51].

### 3.5. Other Effects of Lycopene on Prostate Cancer Cells

Several further *in vitro* studies have been undertaken trying to explain the mechanistic effects of lycopene on prostate cancer. A dose-dependent influence on androgen receptor element expression has been detected in LNCaP cells using a luciferase-reporter assay. It has been shown that lycopene inhibits the androgen receptor element, resulting in decreased PSA velocity, and may, therefore, provide an anti-hormonal potential. However, the effective end-concentration of 15 μmol/L has been supra-physiological [73].

Lycopene might also have an impact on invasion and migration of prostate cancer cells by reducing the expression of integrins, which are known to be involved in signalling processes regarding adhesion and invasion. Bureyko *et al*. [74] reported a decrease of α_2_β_1_-integrin-expression in 22Rv1-, LNCaP- and PC3- cells upon lycopene exposure. α_v_β_3_ and α_v_β_5_ integrins, usually highly expressed in more aggressive prostate cancer cell lines, have been found to be significantly decreased in PC3 cells after treatment with 0.01 μM lycopene. The lycopene concentrations used have been declared to lie within the physiological range [74].

Additionally, lycopene has been shown to inhibit signalling of insulin-like growth factor-I (IGF-I) and, therefore, disrupts one pathway in the development of prostate cancer. Signalling of IGF-I and IGF-II via their receptor, IGF-IR, facilitates survival and proliferation of cancer cells using PI3K/Akt and MAPK pathways. Mobilization of Akt, which plays a key role in inducing IGF-I signals, may have a significant role in the development of an androgen-independent type of prostate-cancer [48]. After exposure of LNCaP cells to lycopene, a reduction of IGF-IR expression, as well as an Akt activation and an increase in the expression of insulin-like growth factor binding protein 2 (IGFBP2) has been shown. IGFBP2 is a binding protein for IGF-I, which has been shown to be highly expressed in LNCaP cells [59]. Another study conducted by Tang *et al*. [75] revealed that lycopene induced a more distinct inhibitory effect on the growth of DU145 prostate cancer cells, which exhibit a greater expression profile of IGF-IR, than on LNCaP, 22Rv1 or PC3 cells, which have a less pronounced IGF-IR level. Though lycopene accumulation in LNCaP cells has been detected to be more than four times higher than in DU145 cells, the growth inhibitory effect has been shown to be seven times less distinct. IGF-IR levels may play an important role in lycopene-mediated inhibition of cancer cell growth and are even more significant than the consideration of lycopene concentrations alone [75]. This study did not only show the involvement of IGF-IR in cell growth inhibition, but also, that the growth of DU-145, LNCaP, 22Rv1 and PC3 cells can further be inhibited by combining docetaxel and lycopene. Again, the greatest effect has been reported for DU-145 cells, exhibiting the highest levels of IGF-IR expression. Combining docetaxel therapy with lycopene may, therefore, especially be effective in cancer types expressing high levels of IGF-IR activity [75].

**Figure 2:**
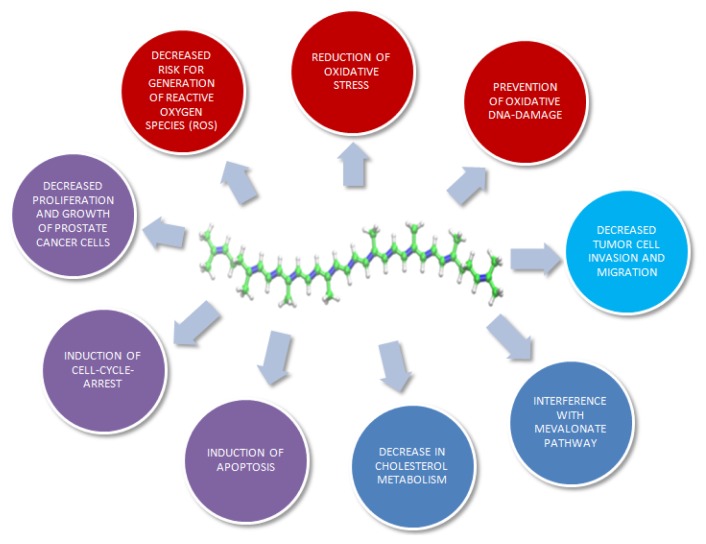
Potential effects of lycopene on prostate cancer cells.

The above mentioned effects of lycopene are summarized in Figure 2. Taken all these effects and results together, a protective role of lycopene in prostate cancer can be embraced. However, the outcomes of the different studies are difficult to interpret and compare, as *in vitro* techniques show wide variations regarding cell lines, culture conditions, lycopene sources, concentrations and solubilizers of lycopene used [60].

As lycopene is very lipophilic, co-solvents, like tetrahydrofuran (THF), foetal bovine serum (FBS) or preparations in special formulations, like water-dispersible beadlets, are necessary to solve and stabilize the molecule. Attempts to improve the availability include also the use of the emulsifiers, Tween 40 and Tween 80, as well as liposomal delivery systems [76–78]. However, every solubilizer has limitations and can influence, to some extent, the outcome of a study. THF, as well as compounds of liposomes, for example, have been shown to have toxic effects on some cell lines, whereas beadlets are composed of ingredients, like ascorbic acid and α-tocopherol, which are able to act as antioxidants by themselves. Tween and FBS have been shown to be less effective in stabilizing lycopene as compared to other solvents [15]. Results indicating no or low effects of lycopene are often conducted in a concentration range at the lower limit, whereas, on the other hand, some studies picture positive outcomes, although the lycopene concentrations used are supra-physiological. Furthermore, the interpretation of different studies is complicated, due to the high variation of lycopene formulations used. Spectra range from naturally derived extracted lycopene over synthetic lycopene formulations to tomato extracts, all containing different active compounds. It is therefore not surprising that cell culture studies investigating lycopene effects show great variations in their outcomes.

## 4. *In Vivo* Models

The first *in vivo* effects of lycopene were reported by Lindberg and co-workers in 1959. The group showed that the ingestion of lycopene led to an increased survival of previously irradiated mice and to a lower incidence of radiation-induced peritoneal tumours [79,80]. Nevertheless, the first animal studies concerning the effects of lycopene on prostate cancer were not performed until the first studies had been published linking lycopene or tomato products with an antitumorigenic effect in prostate cancer patients. Though plenty of clinical studies have been performed so far, only a few preclinical *in vivo* studies have been published (Table 3). In August 2011, Ilic *et al*. identified 64 clinical studies [81], whereas at the same time, only 12 animal models had been published investigating the effects of lycopene on prostate cancer [75,82–90].

In the early 2000s, mainly syngeneic and chemically-induced models of prostate cancer have been used. Imaida *et al*. have been the first to test the chemopreventive efficacy of lycopene using a chemically-induced rat prostate cancer model (six-week-old male F344 rats) [85]. After carcinogen exposure with 3,2′-dimethyl-4-aminobiphenol for 20 weeks, lycopene purified from tomato extracts (99.9%, LycoRed™, Beer-Sheva, Israel) was incorporated in the basal diet (Oriental MF; Oriental Yeast co. Ltd, Tokyo, Japan) at a dosage of 15–45 ppm for 40 weeks. The authors found a significantly decreased incidence of prostatic intraepithelial neoplasia and carcinoma of the ventral prostate in animals treated with lycopene. However, these results have not been reproducible in subsequent experiments [85].

Guttenplan and co-workers examined the effects of dietary supplementation of a lycopene-rich tomato oleoresin on benzo[a]pyrene induced mutagenesis in six-week-old male *lac*Z mice [84]. The supplement consisted mainly of a 3.7% lycopene suspension (69 mM) in medium chain triglycerides (Cognis Corporation, LaGrange, IL, USA). The suspension contained different other natural carotenoids extracted from tomatoes (0.3% phytofluene, 0.44% *Z*-carotene, 0.47% 2,6-cyclolycopene-1,5-diol and 1.2% β-carotene) and was incorporated into an AIN-76A diet at a concentration of 7 and 14 g/kg diet for eight weeks in parallel to carcinogen exposure. Carcinogen-induced tumorigenesis in prostates from the lycopene treated groups was decreased as compared to a control group without lycopene supplementation. However, the differences have not been significant [84].

In 2003, Boileau *et al*. showed that supplementation with water-dispersible beadlets of synthetic lycopene (Hoffman-La Roche, Basel, Switzerland) at a concentration of 2.5 g of beadlets/kg of AIN-93G diet had no effects on the prostate cancer-specific survival of male Wistar rats. To induce carcinogenesis, they were previously treated with cyproterone, testosterone and *N*-methyl-*N*-nitrosourea [82]. However, the authors found that powder derived from heat-processed tomato paste (Armour/Del Monte Foods, San Francisco, CA, USA) incorporated into the diet at the same concentration as the synthetic lycopene was able to increase survival [82].

Siler and co-workers used the MatLyLu-cell line to establish prostate tumours in 8–10-week-old male Copenhagen rats [87]. Supplementation of synthetic lycopene at a dosage of 200 ppm (lycopene 5% TG, DSM Nutritional Products, Basel, Switzerland) incorporated into a standard basal diet (Kliba #2019, Provimi Kliba AG, Kaiseraugst, Switzerland) for four weeks led to a significant increase of necrotic area within the tumours when compared to animals without lycopene supplementation. Hence, the authors suggested that lycopene can lead to a reduction of the prostatic tumour mass [87]. The same group found that synthetic lycopene induces anti-androgen and anti-inflammatory effects, both in cancerous and in healthy prostate tissue [88].

Venkateswaran *et al*. have been the first to use a transgenic mouse model to investigate the effects of lycopene on prostate cancer [89]. They incorporated a mixture of antioxidants containing 800 IU vitamin E, 200 μg selenium and 50 mg lycopene into a standard diet (Purina Mills Test Diet, Richmond, Indiana, USA). After 25 weeks, they euthanized the animals and observed a four-fold reduction in the incidence of prostate cancer compared to untreated mice. Unfortunately, the authors did not provide any information about the formulations of the antioxidants used. The contribution of the different micronutrients was also not evaluated [89].

In 2005 Tang *et al*. [91] analysed the effect of natural lycopene on the growth of androgen-independent human DU145 prostate cancer cells after subcutaneous injection (1 × 10^7^ cells/100 μL Matrigel™) into the flanks of 4–6 week old male BALB/c nude mice. The lycopene-formulation used consisted of >95% pure lycopene with 6% lycopene oleoresin extracted from tomatoes. After tumour cell injection, the mice were gavaged five days per week with different dosages of lycopene (0, 10, 100 and 300 mg/kg body weight) for eight weeks. The authors showed a decrease in tumour growth by 55.6% and 75.8% in mice treated with 100 mg and 300 mg lycopene. Mice injected with DU145 cells pre-treated with 20 μmol/L lycopene did not show any tumour formation after one month [91].

Canene-Adams *et al*. implanted Dunning R3327-H tumour tissue with Matrigel™ (100 mg of tumour/mL Matrigel™) into the flanks of four-week-old male Copenhagen rats [83]. Synthetic lycopene beadlets (lycopene 5% TG, DSM Nutritional Products, Basel, Switzerland) or powder derived from tomatoes (Gilroy Foods, Gilroy, CA, USA) was incorporated into an AIN 93G-based diet, and supplementation started one month prior to tumour tissue transplantation. Tumour analysis at 18 weeks after tumour transplantation showed that 23 nmol or 224 nmol of synthetic lycopene beadlets per gram of diet insignificantly reduced tumour weights, whereas tomato powder at 13 nmol per gram of diet was able to significantly reduce the weight of the tumours as compared to the control groups [83]. In 2010, Konijeti *et al*. [92] used a TRAMP model to compare the effects of lycopene beadlets (lycopene 10%, DSM Nutritional Products, Parsippany, NJ, USA) and tomato paste (Campbell’s Soup Company, Camden, NJ, USA) on the incidence of prostate cancer. Both supplements were incorporated into the basal AIN 93 diet with 100 mg lycopene/kg diet since the age of weaning. In contrast to previously published studies [82,93], the authors found a significantly decreased incidence of prostate cancer in those animals treated with synthetic lycopene, but not in the animals treated with the tomato paste [92].

In 2011, Tang and co-workers [75] investigated the effect of synthetic lycopene and docetaxel on the survival and growth rate of xenografted tumours. DU145 prostate cancer cells were injected at a concentration of 1 × 10^6^ cells/100 μL PBS into the right flank of NCR-*nu/nu* nude mice. After the tumours reached a size of approximately 200 mm^3^, the mice were treated either with 15 mg/kg body weight of synthetic microencapsulated lycopene (LycoVit™ 10% CWD, BASF Corporation, Shreveport, LA, USA) via gavage or with an intraperitoneal injection of docetaxel or with a combination of both. Docetaxel plus lycopene led to a significantly higher tumour regression and increase in survival when compared to docetaxel alone. The synthetic lycopene was able to enhance the antitumor capacity of docetaxel, even when it was given at a suboptimal dosing. However, lycopene alone had no effects on tumour regression or survival [75].

Yang and colleagues implanted human PC3 prostate cancer cells (1 × 10^7^ cells/100 μL PBS) into the flanks of 6–8-week-old athymic nude mice [90]. Subsequently, the mice were gavaged with 4 or 16 mg/kg body weight of lycopene (97%, Wako Pure Chemical Industries, Japan; purified from tomatoes) or 16 mg/kg β-carotene suspended in corn oil twice a day for seven weeks. The authors showed that both lycopene and β-carotene were able to significantly decrease tumour volume and weight when compared to a control group without supplementation. The lycopene effect has been shown to be dosage-dependent [90].

The results of previously published animal studies are in large parts inconsistent. This is mainly due to the heterogeneity of the lycopene formulations and animal models used. As reviewed in Table 3, lycopene formulations vary from synthetic lycopene to purified naturally derived lycopene and non-purified tomato powder or mixtures of different substances. Several other critical issues need to be considered, such as the dosage, form and bioavailability of the active component and the timing of the treatment. Nevertheless, the preclinical data strongly suggests an antitumorigenic activity of lycopene and its different formulations, either alone or in combination with other substances.

## 5. Lycopene for the Prevention and Therapy of Prostate Cancer: Clinical Evidence

Lycopene, with its abundant availability, low costs and lack of side effects, would be a suitable antitumorigenic drug, but there is still no clear clinical evidence whether to support or refute its use for the prevention or therapy of prostate cancer [94]. In the literature, there are plenty of epidemiological studies [95–98] analysing the effects of tomato-based products on prostate cancer, but research that specifically investigates lycopene supplementation is limited. Most of the published studies have a low level of evidence, and there is only a limited number of published randomized clinical trials [99].

A Cochrane review published in 2011 by Ilic and co-workers [81] investigated the capability of lycopene for the prevention of prostate cancer and identified only three randomized controlled trials with a total of 154 participants with prostatic intraepithelial neoplasia or benign prostate hyperplasia using lycopene as the intervention. Two [100,101] of these three studies [100–102] posed a high risk of bias, especially in blinding of participants and personnel [81]. Only one of these three studies reported the incidence of prostate cancer. Although a decreased prostate cancer incidence has been found for the intervention group, the difference to the control group was statistically not significant [101].

In 2011, van Breemen *et al*. [103] published the results of another randomized controlled clinical trial including 105 men with a PSA level greater than 4 ng/mL and abnormal digital rectal examination or ultrasonography. Twenty-one days before, prostate biopsy patients were either randomized to the lycopene or the control group. Effects on PSA level or incidence of prostate carcinoma have not been reported in this study. Lycopene concentration in plasma and prostate tissue from the patients who received lycopene has been significantly higher than in the control group. However, no significant differences in molecular markers of oxidative stress could be found [103] (Table 4).

Though there is a considerable interest in lycopene as a therapeutic agent, only few high quality studies have been reported analysing its effect on prostate cancer. In 2012, Ilic and Misso [99] published a systematic review in which they identified only four randomized controlled clinical studies that tested lycopene as a therapeutic agent for prostate cancer (Table 5) [104–107]. One of the studies posed a high risk of bias [105].

## 6. Conclusions

While *in vitro* studies predominantly demonstrated the chemopreventive effects of lycopene on prostate cancer cells, its effectiveness *in vivo* has still to be proven. Attempts to recapitulate the antitumorigenic activity of lycopene in animal models have been in some parts highly significant and showed interesting data, suggesting the potential of lycopene to reduce tumour growth rate and to increase the survival of the animals. However, the results have mainly been inconsistent [108]. Stringent animal models and a clear definition of the lycopene-preparations used are strongly recommended before results can be transferred to the clinical setting. Nevertheless, the use of lycopene in the clinic still rests on findings derived from randomized controlled clinical trials. Recent systematic reviews could show that lycopene is able to decrease the serum PSA-levels in patients with prostate hyperplasia or cancer, demonstrating its effect on proliferating prostate cells [81,99]. In the future, newly designed randomized controlled clinical trials might contribute to a better understanding of lycopene effects on prostate cancer patients.

## Figures and Tables

**Table 1:** Lycopene content of common fruits and vegetables [16,26,27].

Lycopene content [mg/100 g wet basis]	Source
0.7–20	Fresh tomatoes
3.7	Cooked tomatoes
2.3–7.2	Fresh watermelon
2.0–5.3	Fresh papaya
5.2–5.5	Pink guava
0.4–3.4	Pink grapefruit
0.01–0.05	Apricot

**Table 2:** Lycopene content of various tomato products (according to Tonucci *et al*. [33]).

Lycopene content (mg/100 g) ± SD	Source
9.27 ± 1.02	Raw tomatoes
17.23 ± 2.18	Ketchup
15.99 ± 0.90	Spaghetti sauce
55.45 ± 4.33	Tomato paste
16.67	Tomato puree
17.98 ± 1.47	Tomato sauce

**Table 3:** Animal models investigating the effect of lycopene on prostate cancer.

Author	Year	Strain	Tumour model	Lycopene formulation and source	Outcome
Imaida *et al*. [85]	2001	6-week-old male F344 rats	Chemically induced with 3,2′-dimethyl-4-aminobiphenol	Lycopene purified from tomato extracts (99.9%, LycoRed™, Beer-Sheva, Israel)	Significantly decreased incidence of prostatic intraepithelial neoplasia and carcinoma of the ventral prostate
Guttenplan *et al*. [84]	2001	6-week-old male *lac*Z mice	Chemically induced with benzo[a]pyrene	Suspension of 3.7% Lycopene, 0.3% phytofluene, 0.44% *Z*-carotene, 0.47% 2,6-cyclolycopene-1,5-diol and 1.2% β-carotene extracted from tomatoes (Cognis Corporation, LaGrange, IL, USA)	Carcinogen-induced mutagenesis in prostates from the lycopene treated groups was decreased compared to a control group without lycopene supplementation
Boileau *et al*. [82]	2003	5-week-old male Wistar rats	Chemically induced with cyproterone, testosterone and *N*-methyl-*N*-nitrosourea	Beadlets of synthetic lycopene (Hoffman-La Roche, Basel, Switzerland) or powder derived from heat-processed tomato paste with skin and seeds (Armour/Del Monte Foods, San Francisco, CA, USA)	Tomato powder, but not synthetic lycopene, was able to increase prostate cancer-specific survival
iler *et al*. [87]	2004, 2005	8–10-week-old male Copenhagen rats	Orthotopic implantation of MatLyLu cells into the ventral prostate	Beadlets of synthetic lycopene (Lycopene 5% TG, DSM Nutritional Products, Basel, Switzerland)	MRI revealed a significant increase of necrotic area in tumours of lycopene treated animals. Anti-androgen and anti-inflammatory effects on cancerous and healthy prostate tissue
Venkateswaran *et al*. [89]	2004	4–5-week-old transgenic male *Lady* mice	Transgenic adenocarcinoma of the mouse prostate (TRAMP) model	Mixture of antioxidants containing 800 IU vitamin E, 200 μg Selenium and 50 mg lycopene (no information about the supplier or the formulation was given)	The mixture of micronutrients was able to inhibit prostate cancer development and to increase the disease-free survival of the animals.
Tang *et al*. [91]	2005	4–6-week-old male BALB/c nude mice	Subcutaneous xenotransplantation of human prostate cancer cells (DU-145) along with Matrigel™	>95% pure lycopene with 6% lycopene oleoresin purified from tomato extracts (no information about the supplier was given)	Decrease in tumour growth by 55.6% and 75.8% in mice treated with 100 mg and 300 mg lycopene, respectively
Canene-Adams *et al*. [83]	2007	4-week-old male Copenhagen rats	Subcutaneous implantation of minced Dunning R3327-H tumour tissue suspended in Matrigel™	Beadlets of synthetic lycopene (Lycopene 5% TG, DSM Nutritional Products, Basel, Switzerland) or powder derived from tomatoes (Gilroy Foods, Gilroy, CA, USA)	Synthetic lycopene insignificantly reduced tumour weight, whereas the reduction of tumour weight was significant for tomato powder.
Konijeti *et al*. [92]	2010	male transgenic TRAMP mice at weaning age	TRAMP model	Beadlets of synthetic lycopene (Lycopene 10%, DSM Nutritional Products, Parsippany, NJ, USA) or tomato paste without skin and seeds (Campbell’s Soup Company, Camden, NJ, USA)	Prostate cancer incidence was significantly decreased in the lycopene beadlet group, whereas the difference between the tomato paste group and the control group was not significant.
Tang *et al*. [75]	2011	NCR-*nu/nu* mice (no information about the age)	Subcutaneous xenotransplantation of human prostate cancer cells (DU-145)	Microencapsulated synthetic lycopene (LycoVit™ 10% CWD, BASF Corporation, Shreveport, LA, USA) alone or in combination with docetaxel i.p.	Synthetic lycopene alone had no significant effects on tumour regression or survival. Docetaxel plus lycopene led to a significant tumour regression and increase in survival when compared to docetaxel alone.
Yang *et al*. [90]	2011	6–8-week-old male athymic nude mice	Subcutaneous xenotransplantation of human prostate cancer cells (PC-3)	Lycopene purified from tomato extracts (97%, Wako Pure Chemical Industries, Japan) or β-carotene	Both lycopene and β-carotene significantly decreased tumour volume and weight.

**Table 4:** Randomized controlled clinical trials analysing the effects of lycopene on the prevention of prostate cancer.

Author	Year	Patients	Follow-up	Lycopene formulation and source	Outcome
Mohanty *et al*. [101]	2005	40 patients with high-grade intraepithelial neoplasia (intervention group: 20, control group: 20)	2 years	4 mg lycopene (Lyc-O-Mato™, LycoRed Natural Products Industries, Ltd., Beer-Sheva, Israel) twice a day for one year; Lyc-O-Mato™ is a tomato-oleoresin extracted from tomatoes, which contains a high amount of lycopene as well as other natural tomato phytonutrients, such as tocopherols, phytoene, phytofluene, β-carotene, phospholipids and phytosterols.	The incidence of prostate cancer was 10% in the intervention and 30% in the control group. PSA levels in the lycopene group decreased from a mean level of 6.07 to 3.5 ng/mL and increased in the control group from 6.55 to 8.06 ng/mL. Accordingly, the serum lycopene increased in the treatment group from 360 to 680 ng/mL and decreased in the control group from 378 to 180 ng/mL.
Bunker *et al.* [100]	2007	80 patients with high-grade intraepithelial neoplasia, atypical foci or more than one non-cancerous biopsy (intervention group: 40, control group: 40; both groups were treated with a multivitamin mixture during the study)	4 months	15 mg lycopene (Lyc-O-Mato™, LycoRed Natural Products Industries, Ltd., Beer-Sheva, Israel) twice a day for the duration of the follow-up	No difference in PSA levels between groups were reported, neither at one month, nor four months after initiation of the intervention. Serum lycopene levels doubled in the intervention group. Incidence of prostate cancer was not reported.
Schwarz *et al.* [102]	2008	40 patients with histologically proven benign prostate hyperplasia (intervention group: 20, control group: 20)	6 months	15 mg microencapsulated synthetic lycopene (LycoVit™ 10%, BASF, Ludwigshafen, Germany) once a day for the duration of the follow-up	Significant reduction of PSA levels in the intervention group, but not in the control group (*p* < 0.05). Accordingly, serum lycopene was increased in the intervention, but not in the control group (*p* < 0.0001). Prostate enlargement could be detected in the control group via trans-urethral ultrasonography (*p* < 0.05) and digital rectal examination (*p* < 0.01), whereas the prostate did not enlarge in the intervention group. Incidence of prostate cancer was not reported.
Van Breemen *et al.* [103]	2011	131 patients scheduled for prostate cancer biopsy as a result of elevated PSA levels and an abnormal digital rectal examination or ultrasonography (intervention group: 69, control group 62)	21 days	15 mg lycopene (Lyc-O-Mato™, LycoRed Natural Products Industries, Ltd., Beer-Sheva, Israel) twice a day for 21 days	Significant increase of lycopene levels in the serum (*p* < 0.0001) and prostate tissue (*p* = 0.005) in the intervention group. No significant changes in the DNA oxidation product 8-oxo-desoxyguanosine and the lipid peroxidation product, malondialdehyde, in prostate tissue or plasma. No significant difference in prostate cancer incidence.

**Table 5:** Randomized controlled clinical trials analysing the therapeutic effects of lycopene in prostate cancer.

Author	Year	Patients	Follow-up	Lycopene formulation and source	Outcome
Kucuk *et al*. [106]	2001	26 patients with clinically diagnosed prostate cancer prior to scheduled prostatectomy (intervention group: 15, control group: 11)	3 weeks	15 mg lycopene (Lyc-O-Mato™, LycoRed Natural Products Industries, Ltd., Beer-Sheva, Israel) once a day for 3 weeks	PSA serum levels decreased by 18% in the intervention group and increased by 14% in the control group (*p* = 0.25). 73% of the intervention and 18% of the control group had microscopically free resection margins (*p* = 0.02). 84% of the intervention and 45% of the control group had tumour volumes >4 mL (*p* = 0.222).
Ansari et Gupta [104]	2003	54 patients with prostate cancer metastases and orchidectomy (intervention group: 27, control group: 27)	24–28 months	2 mg lycopene twice a day (no information about the supplier or the formulation)	After 2 years, the PSA serum levels were significantly lower in the intervention group (*p* < 0.001). The survival rate measured by the Kaplan-Meier method was significantly higher in the intervention group (*p* < 0.001). There was a significantly improvement in urinary peak flow rate in the lycopene group (*p* = 0.04)
Vaishampayan *et al*. [107]	2007	71 patients with histologically proven prostate cancer and rising serum PSA levels following local therapy or during hormone therapy (intervention group: 38, control group: 33)	5.5–6 months	15 mg lycopene (Lyc-O-Mato™, LycoRed Natural Products Industries, Ltd., Beer-Sheva, Israel) twice a day for 6 months in the intervention group. The control group was treated with the same dosage of lycopene and soy isoflavone 40 mg twice a day for 5.5 months.	No decline in serum PSA level in either group. However, in both therapeutic arms, there was a significant decline in the rate of PSA increase from pre-therapy to post-therapy.
Grainger *et al*. [105]	2008	41 patients with recurrent prostate cancer (intervention group: 20, control group: 21)	8 weeks	The diet in the intervention group consisted of tomato and/or tomato products containing at least 25 mg lycopene per day given for 4 weeks. The control group was treated with soy protein. After 4 weeks, both groups were treated with lycopene 25 mg and soy protein for another 4 weeks.	Serum lycopene levels increased significantly in both groups after 8 weeks of dietary intervention. There was no statistically significant difference in PSA serum levels between the groups.
